# Beyond genomics: artificial intelligence-powered diagnostics for indeterminate thyroid nodules—a systematic review and meta-analysis

**DOI:** 10.3389/fendo.2025.1506729

**Published:** 2025-05-05

**Authors:** Karishma Jassal, Melissa Edwards, Afsaneh Koohestani, Wendy Brown, Jonathan W. Serpell, James C. Lee

**Affiliations:** ^1^ Monash University Endocrine Surgery Unit, Alfred Hospital, Melbourne, VIC, Australia; ^2^ Department of Surgery, Central Clinical School, Monash University, Melbourne, VIC, Australia

**Keywords:** artificial intelligence, thyroid cancer, thyroid nodule - diagnosis, meta - analysis, machine learning

## Abstract

**Introduction:**

In recent years, artificial intelligence (AI) tools have become widely studied for thyroid ultrasonography (USG) classification. The real-world applicability of these developed tools as pre-operative diagnostic aids is limited due to model overfitting, clinician trust, and a lack of gold standard surgical histology as ground truth class label. The ongoing dilemma within clinical thyroidology is surgical decision making for indeterminate thyroid nodules (ITN). Genomic sequencing classifiers (GSC) have been utilised for this purpose; however, costs and availability preclude universal adoption creating an inequity gap. We conducted this review to analyse the current evidence of AI in ITN diagnosis without the use of GSC.

**Methods:**

English language articles evaluating the diagnostic accuracy of AI for ITNs were identified. A systematic search of PubMed, Google Scholar, and Scopus from inception to 18 February 2025 was performed using comprehensive search strategies incorporating MeSH headings and keywords relating to AI, indeterminate thyroid nodules, and pre-operative diagnosis. This systematic review and meta-analysis was conducted in accordance with methods recommended by the Cochrane Collaboration (PROSPERO ID CRD42023438011).

**Results:**

The search strategy yielded 134 records after the removal of duplicates. A total of 20 models were presented in the seven studies included, five of which were radiological driven, one utilised natural language processing, and one focused on cytology. The pooled meta-analysis incorporated 16 area under the curve (AUC) results derived from 15 models across three studies yielding a combined estimate of 0.82 (95% CI: 0.81–0.84) indicating moderate-to-good classification performance across machine learning (ML) and deep learning (DL) architectures. However, substantial heterogeneity was observed, particularly among DL models (I² = 99.7%, pooled AUC = 0.85, 95% CI: 0.85–0.86). Minimal heterogeneity was observed among ML models (I² = 0.7%), with a pooled AUC of 0.75 (95% CI: 0.70–0.81). Meta-regression analysis performed suggests potential publication bias or systematic differences in model architectures, dataset composition, and validation methodologies.

**Conclusion:**

This review demonstrated the burgeoning potential of AI to be of clinical value in surgical decision making for ITNs; however, study-developed models were unsuitable for clinical implementation based on performance alone at their current states or lacked robust independent external validation. There is substantial capacity for further development in this field.

**Systematic Review Registration:**

https://www.crd.york.ac.uk/PROSPERO/, identifier CRD42023438011.

## 1 Introduction

The prevalence of incidentally detected thyroid nodules in adults is estimated to be between 30% and 70%, the majority of which are inconsequential, and only approximately 5% are ultimately proven to be malignant (1–4). Evaluation of nodules conventionally begins with ultrasonography (USG) where standardised acquisition of radiological features in accordance to one of several Thyroid Image Reporting and Data Systems (TIRADS) leads to further diagnostic steps (5–7). Fine-needle aspiration cytology (FNAC) subsequently facilitates the categorisation of thyroid nodules as malignant, benign, or indeterminate according to the six-tiered Bethesda classification (8). Whilst studies have shown that 95% of samples are adequate for interpretation, 20%–25% of aspirates are reported as indeterminate (Bethesda categories III–V), with substantial variability in the probability of malignancy within this category (9–11).

Standard strategies for clarifying the diagnosis are either diagnostic thyroid lobectomy or repeating FNAC typically for Bethesda III lesions at 3 months from the initial procedure to allow for the resolution of inflammatory changes, which is a safe procedure and a practical approach (8, 12). Clinical and sonographic considerations are recommended when electing for repeat sampling and, in the majority of cases, do not lead to diagnostic resolution potentially risking delaying treatment of malignancy (8, 12–14). The important caveat in real clinical practice is that the patient still needs to be informed of the highest implied malignancy risk of any FNAC sample, which can lead to confusion and anxiety. Diagnostic lobectomy requires multiple considered steps to preserve parathyroid and recurrent laryngeal nerve function, in addition to the risks of haematoma, infection, and post-operative hypothyroidism (15–18). Patients with malignancy may subsequently require a second-stage operation for completion of surgical treatment, which can be more technically challenging due to post-operative tissue changes.

More recently, genomic sequencing classifiers (GSC) have been utilised to interrogate indeterminate cytology thyroid nodules (ITNs). GSC displays high specificity and allows avoiding diagnostic surgery in up to 61% of patients on the basis of a benign test (19–22). This enables a more accurate pre-operative assessment of ITNs. However, the tests are costly, requires additional samples taken, and are not available in many countries. These barriers preclude the universal adoption of GSC, and as such, hemithyroidectomy remains a key diagnostic tool.

Developments in computational technology have led to the development of artificial intelligence (AI) tools beyond GSC that may be useful in thyroid nodule diagnostics. AI tools in thyroid nodule diagnosis are mostly reported using a single diagnostic modality, such as ultrasonographic or cytological characteristics (23–27). These single-entity tools tend to have functionality within a particular branch of medicine, but the question remains if they are applicable within surgical decision making where the process is multifaceted.

We therefore sought to conduct a systematic review and meta-analysis to appraise the available evidence related to the pre-operative diagnostic accuracy of AI tools for indeterminate cytology thyroid nodules, excluding GSC.

## 2 Materials and methods

This systematic review and meta-analysis was conducted in accordance with methods recommended by the Cochrane Collaboration and registered with the International Prospective Register of Systematic Reviews (PROSPERO), reference no. CRD42023438011 (28). Reporting follows the standards of the Preferred Reporting Items for Systematic Reviews and Meta-analysis Statement (PRISMA) (29, 30).

### 2.1 Search strategy

English language articles evaluating the diagnostic accuracy of AI for ITNs were identified. A systematic search of PubMed, Google Scholar, and Scopus from inception to 18 February 2025 was performed using comprehensive search strategies incorporating MeSH headings and keywords relating to AI, indeterminate thyroid nodules, and diagnosis [Boolean string; preop* AND (diagno* OR evaluat*) AND (“artificial intelligence” OR “machine learning”) AND “indeterminate thyroid nodules”—molecular]. An additional search was conducted specifically to target cytology-based studies [Boolean string; *(“thyroid nodule/pathology” OR “biopsy, fine-needle/methods”) AND (“artificial intelligence” OR “machine learning”)].* Screening on the title was performed until saturation, which was reached at 50 studies. The papers in the reference lists of included articles and relevant reviews were reviewed to identify additional eligible publications. The inclusion criteria for this review were developed in accordance with the following PICO framework: Can pre-operative patients with ITNs (P) be evaluated using AI models to predict malignancy (I) in terms of diagnostic accuracy measures (O), compared to standard reference diagnoses, such as final histopathology or other established diagnostic methods (C), excluding studies involving GSC? Both randomised and non-randomised studies were included. Qualitative studies, abstracts, reviews, editorials, and case studies were excluded.

As Bethesda III–V nodules are usually managed similarly surgically, the search targeted articles, which included adult patients with ITNs (Bethesda categories III–V on FNAC) who underwent surgery. “Artificial intelligence” was defined as a machine learning (ML) or deep learning (DL) tool that identifies patterns resulting in a prediction. Application of any type of AI models, including classifiers, neural networks, or natural language processing (NLP), was accepted (31). Both model development and validation studies were included. Only studies that provided a clear distinction between benign and malignant prediction outcomes were considered. For studies that reported results based on histological subtypes or other stratifications, only outcomes relevant to benign and malignant classification were extracted for statistical analysis. Where a study included patients with all Bethesda categories, only outcomes relating to those with indeterminate cytology were considered. The primary outcome measure was model performance, including diagnostic accuracy, area under the curve (AUC), sensitivity, specificity, positive predictive value (PPV), and negative predictive value (NPV).

### 2.2 Data abstraction

Titles and abstracts were independently and manually screened by two reviewers (KJ and ME) using explicit pre-determined criteria. Inconsistencies were resolved through consultation with a third reviewer (JL). Data were extracted from each eligible study by one reviewer (KJ) using a standardised electronic form.

### 2.3 Risk of bias assessment

The Prediction model Risk Of Bias Assessment Tool (PROBAST), used to evaluate the risk of bias (ROB) and applicability of diagnostic and prognostic prediction model studies, was used to assess the included studies (32, 33). ROB and concerns regarding applicability were evaluated with respect to the randomisation process, appropriateness of inclusion/exclusion criteria of participants, assessment of predictors of models created, completeness of outcome data, and model analysis. Overall, ROB was judged as low if all domains assessed returned a low-risk result.

### 2.4 Data synthesis and analysis

Narrative synthesis was used to summarise the main outcomes of interest. Meta-analysis was performed where three or more models assessing a specific outcome measure with an estimate of precision were included. With these criteria, meta-analysis of the area under the curve (AUC) was possible. Statistical analysis was performed using the metan estimation package from Stata/IC for Windows, version 14.2. Given the variability in study designs, random effect models were applied. A value of p < 0.05 was considered statistically significant. Heterogeneity was assessed using Cochran’s Q test (Chi-square test) and quantified using I². Meta-regression was conducted using weighted least squares regression, with standard error (SE) of AUC as the predictor and inverse variance (1/SE²) as weights. Publication bias was evaluated using Egger’s test. A funnel plot was generated using the metafunnel estimation package.

## 3 Results

### 3.1 Study selection

**Figure 1:**
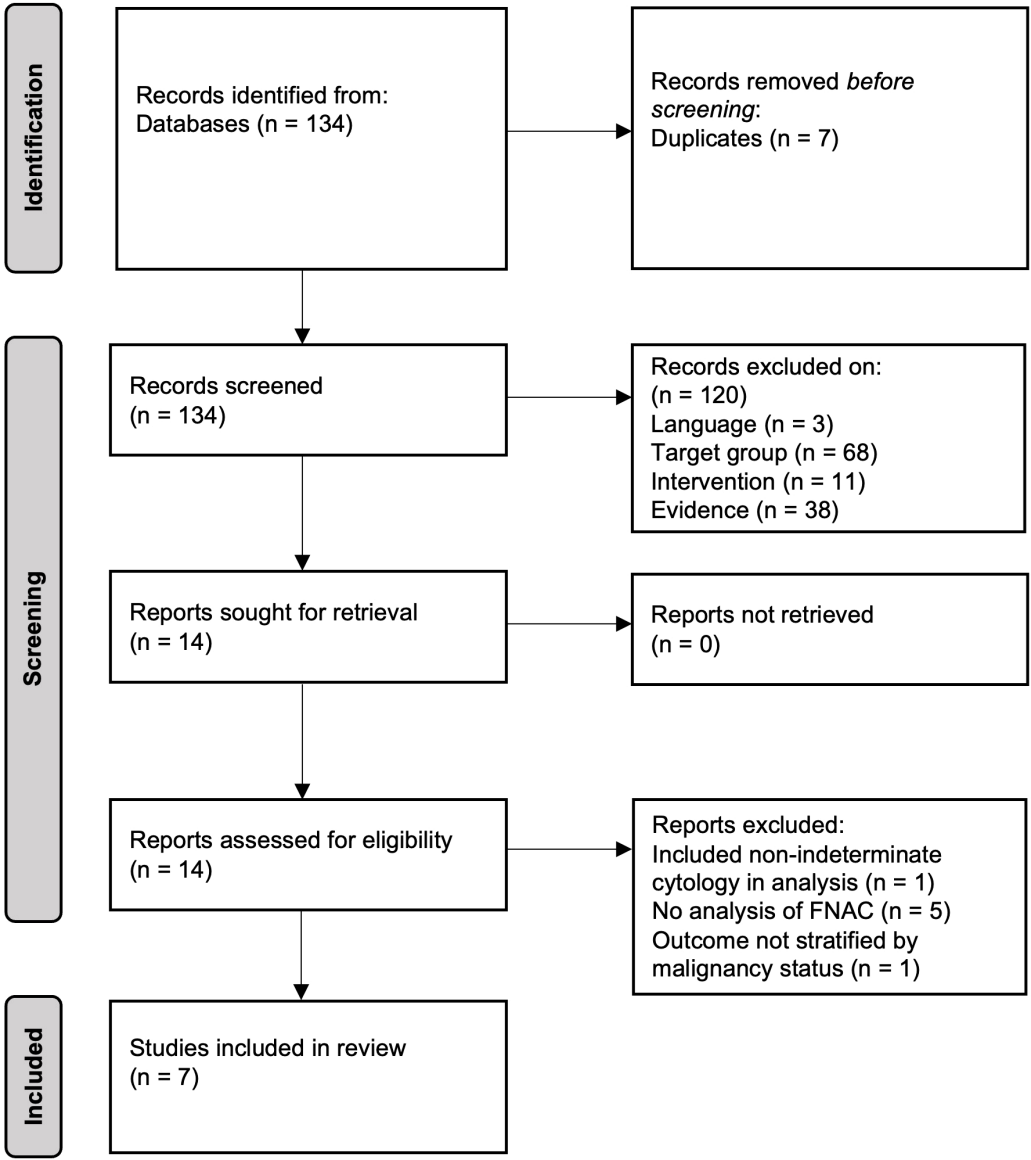
PRISMA flowchart of identification of studies.

The search strategy yielded 134 records after removal of duplicates. Fourteen papers were identified for full text assessment with seven studies meeting the criteria for inclusion in the systematic review (PRISMA flowchart of study selection shown in **Figure 1**). A summary of results from the included studies and models is given in **Table 1**.

**Table 1:** Summary of study characteristics and results.

Author	Subgroup	Bethesda category	Model	Evaluation	Dataset	N	Accuracy	Sensitivity	Specificity	PPV	NPV	AUC 95% CI
Gild	ML	III	RF	–	SI—Internal	88	–	–	–	–	–	0.750 (0.620–0.840)
Gild	DL	III	ResNet-50	10-fold cross validation	SI—Internal	88	74.0	82.0	59.0	56.0	84.0	0.740 (0.590–0.830)
Gild	DL	III	ThyNet	Direct classification	SI—External	88	64.0	–	–	–	–	–
Swan	DL	III, IV, V	AIBx - ResNet, ResNext, DenseNet ensemble	Direct classification	SI—External	155	53.0	96.3	50.0	27.9	81.5	–
Keutgen	ML	III, IV, V	BANN	5-fold cross validation	SI—Internal	19	–	–	–	–	–	0.880 (0.700–1.060)
Keutgen	ML	III, IV, V	BANN	Direct classification	SI—External	20	–	–	–	–	–	0.680 (0.460–0.900)
Luong	ML	III, IV, V	RF	10-fold cross validation	MC—Internal	355	79.1	75.5	82.4	80.3	79.0	0.859 (0.700–0.970)
Luong	ML	III, IV, V	KNN	10-fold cross validation	MC—Internal	355	64.4	52.7	75.3	66.9	63.5	0.664 (0.460–0.800)
Luong	ML	III, IV, V	Ridge	10-fold cross validation	MC—Internal	355	65.7	58.6	72.3	66.6	58.5	0.694 (0.480–0.850)
Luong	ML	III, IV, V	GNB	10-fold cross validation	MC—Internal	355	61.4	30.9	89.8	74.2	58.5	0.694 (0.520–0.870)
Luong	ML	III, IV, V	SVM	10-fold cross validation	MC—Internal	355	63.1	60.8	65.3	62.2	64.5	0.683 (0.490–0.840)
Luong	ML	III, IV, V	ET	10-fold cross validation	MC—Internal	355	74.8	70.5	78.8	76.0	74.8	0.832 (0.660–0.940)
Luong	ML	III, IV, V	AB	10-fold cross validation	MC—Internal	355	72.1	65.1	78.7	74.5	71.2	0.778 (0.620–0.910)
Luong	ML	III, IV, V	GB	10-fold cross validation	MC—Internal	355	77.7	74.5	80.7	78.7	77.8	0.830 (0.680–0.950)
Saini	ML	III	ANN	Direct classification	MC—Internal	11	–	100	100	–	–	1.000 (0.540–1.000)
Chen	ML	III, IV, V	SVM	5-fold cross validation	SI—Internal	194	71.8	93.8	56.5	60	92.9	–
Yao	DL	IV	ResNet50	10-fold cross validation	MC—Internal	1670	79.1	86.5	65.8	81.5	74.5	0.803 (0.794–0.812)
Yao	DL	IV	RadImageNet	10-fold cross validation	MC—Internal	1670	81.6	85.4	69.3	84.0	77.2	0.836 (0.830–0.842)
Yao	DL	IV	ThyNet	10-fold cross validation	MC—Internal	1670	80.4	88.7	69.9	83.4	73.8	0.840 (0.834–0.846)
Yao	DL	IV	Swin Transformer	10-fold cross validation	MC—Internal	1670	90.8	92.7	89.6	93.9	85.9	0.935 (0.929–0.941)

ML, machine learning; DL, deep learning; RF, random forest; BANN, Bayesian artificial neural network; KNN, K-Nearest Neighbour; GNB, Gaussian Naïve Bayes; SVM, support vector machine; ET, Extra Trees; AB, AdaBoost; GB, gradient boosting; SI, single institution; MC, multicentre.

### 3.2 Study characteristics

A total of 20 models were presented in the seven studies included. Of these, 17 models from six studies were independently developed by the corresponding research groups (34–38), and two studies (34, 39) presented external evaluations of previously constructed models without additional pretraining (40, 41). Five studies (34, 36, 37, 39, 42) in this review utilised USG images or characteristics, one study (35) employed an NLP approach, and one study (38) focused on cytological analysis.

### 3.3 Model outcome measures

Five studies based their outcome measures on surgical histopathology sourced from previously established databases (34, 36, 37, 39, 42). One study utilised histopathology to determine malignant outcomes, while a combination of histopathology and close follow-up was used for benign diagnoses (38). In the remaining study, a previously validated clinical NLP software (Apache cTAKES) extracted data from electronic medical record pathology reports to determine outcomes (35, 43). Performance metrics of most models were reported using standardised classification metrics, namely, AUC, accuracy, sensitivity, specificity, PPV, and NPV.

### 3.4 Imaging-based models

Two previously developed USG recognition models were externally evaluated without institution-specific fine-tuning, retraining, or adaptation in separate studies. Gild et al. (34, 36) tested ThyNet’s performance on their patient dataset. ThyNet is a DL network with a reported accuracy of 89.1% in its original study (41). ThyNet achieved an overall accuracy of 64% in this external evaluation (34). Swan et al. (39) retrospectively analysed the performance of AIBx (40) on Bethesda III–V nodules. AIBx is a USG image similarity AI model for the risk stratification of thyroid nodules. The external evaluation of AIBx vs. European Thyroid Association TIRADS for ITNs reports an accuracy of 53.0% vs. 32.2%, PPV of 27.9% vs. 25.2%, NPV of 81.5% vs. 91.7%, sensitivity of 96.3% vs. 63.0%, and specificity of 50.0% vs. 12.5% (39).

One study (34) tested the performance of their two trained models: an image classification convolutional neural network (CNN) utilising the ResNet-50 (44) architecture and a random forest (RF) classifier for first-order statistics of extracted radiomic features. Only Bethesda III nodules were included. The reported AUC for internal validation of the CNN model was 0.74 and 0.75 for the RF radiomics model.

Similarly, Keutgen et al. (42) extracted radiomics features from thyroid nodule USG images obtained from two institutions and utilised a two-class Bayesian artificial neural network classifier to predict the final surgical histopathology of indeterminate cytology nodules. Internal validation results demonstrated an AUC of 0.88 for malignant vs. benign classification and 0.68 on external validation.

A study by Yao et al. (36) evaluated multiple AI models for diagnosing Bethesda IV nodules using USG imaging data collected from five hospitals. Four AI models were trained using a transfer-learning approach, including Swin Transformer, ThyNet, RadImageNet, and ResNet-50 to predict histological outcomes of follicular thyroid cancer (FTC) vs. follicular variant papillary thyroid cancer (FVPTC) vs. benign nodules (41, 45). Model performance was consistent across test sets and 10-fold cross validation, with Swin Transformer achieving the highest AUC (0.917–0.945). PPV and NPV were 93.9% and 85.9%, respectively.

Chen et al. (37) trained a support vector machine (SVM) classifier to distinguish benign nodules from malignant ones utilising five ultrasound input parameters along with nodule size, patient age, and sex. Two radiologists, blinded to clinical and histopathological outcomes, independently reviewed and scored the ultrasound features according to the American College of Radiology TIRADS (ACR TIRADS) criteria—composition, echogenicity, shape, margin, and echogenic foci. A third senior radiologist resolved any disagreements. The model achieved a sensitivity of 93.8%, with a specificity of 56.5%. The NPV for Bethesda III and IV nodules was 93.9% and 93.8%, respectively. Compared to the 2017 ACR TIRADS, the SVM model demonstrated superior performance in distinguishing benign ITNs.

### 3.5 Natural language-processing models

A study by Luong et al. (35) utilised a previously validated NLP model, the Mayo clinical text analysis and knowledge extraction system (cTAKES) (43), to construct several classifier models. This retrospective study included 355 Bethesda III–V nodules from adult patients investigating the utility of cTAKES NLP analysis of readily available electronic medical records (EMR) in predicting malignancy for ITNs. Features extracted from the EMR were age of first FNAC, nodule diameter, height, width, echogenicity, presence of calcification on USG, FNAC results, “largest dimension on cytology,” race, and sex.

The performance of the following eight classifiers were evaluated: Gradient Boosting, SVM, Ridge, Gaussian Naïve Bayes, K-Nearest Neighbour, RF, Extra Trees, and AdaBoost. On average, the accuracy of the classifiers tested was 70.0%, sensitivity 61.1%, specificity 77.9%, PPV 72.4%, NPV 69.4%, and AUC 0.754. The RF classifier performed the best overall, with an accuracy of 79.1%, sensitivity of 75.5%, specificity of 82.4%, PPV of 80.3%, NPV of 79.0%, and AUC of 0.859. The K-Nearest Neighbour classifier produced the least successful results with 64.4% accuracy, 52.7% sensitivity, 75.3% specificity, 66.9% PPV, 63.5% NPV, and 0.664 AUC.

### 3.6 Cytology-based models

Saini et al. developed an artificial neural network (ANN) model to predict the risk of malignancy in Bethesda category III nodules based on FNAC features. Cytological features were subjectively graded by two independent observers and used as input parameters within the ANN that was constructed for binary classification. The features assessed included nuclear pleomorphism, microfollicle formation, nuclear grooving, intranuclear inclusions, nucleoli prominence, Hurthle cell changes, colloid presence, cellularity, and nuclear chromatin characteristics. Each parameter was graded on a semi-quantitative scale from zero to three based on its prevalence in the smear. The model successfully classified all benign and malignant cases within the study’s test set, with an AUC of 1, indicating perfect discrimination (38).

### 3.7 Meta-analysis

#### 3.7.1 Model variability and generalisation challenges

Dissimilarities between studies were acknowledged and accepted to reach a unified conclusion. A fundamental challenge in AI applications is the lack of generalisability, as many models demonstrate high accuracy in controlled environments but underperform when applied to diverse clinical settings. Given that AI in medical diagnostics is still in a relatively early phase of development, there is considerable experimentation with a wide range of models and methodologies. Consequently, the studies included in this review employed a variety of AI algorithms, with notable differences in their training and validation processes. Although this heterogeneity may initially appear to be a limitation, it is reflective of the ongoing iterative process of AI development. Moreover, this diversity strengthens our analysis by providing a more comprehensive evaluation of AI model performance across different contexts. By integrating these disparate results, we gain a broader understanding of the current capabilities and limitations of AI in the pre-operative diagnosis of indeterminate thyroid nodules, which is essential for guiding future research and development.

#### 3.7.2 Pooled analysis

**Figure 2:**
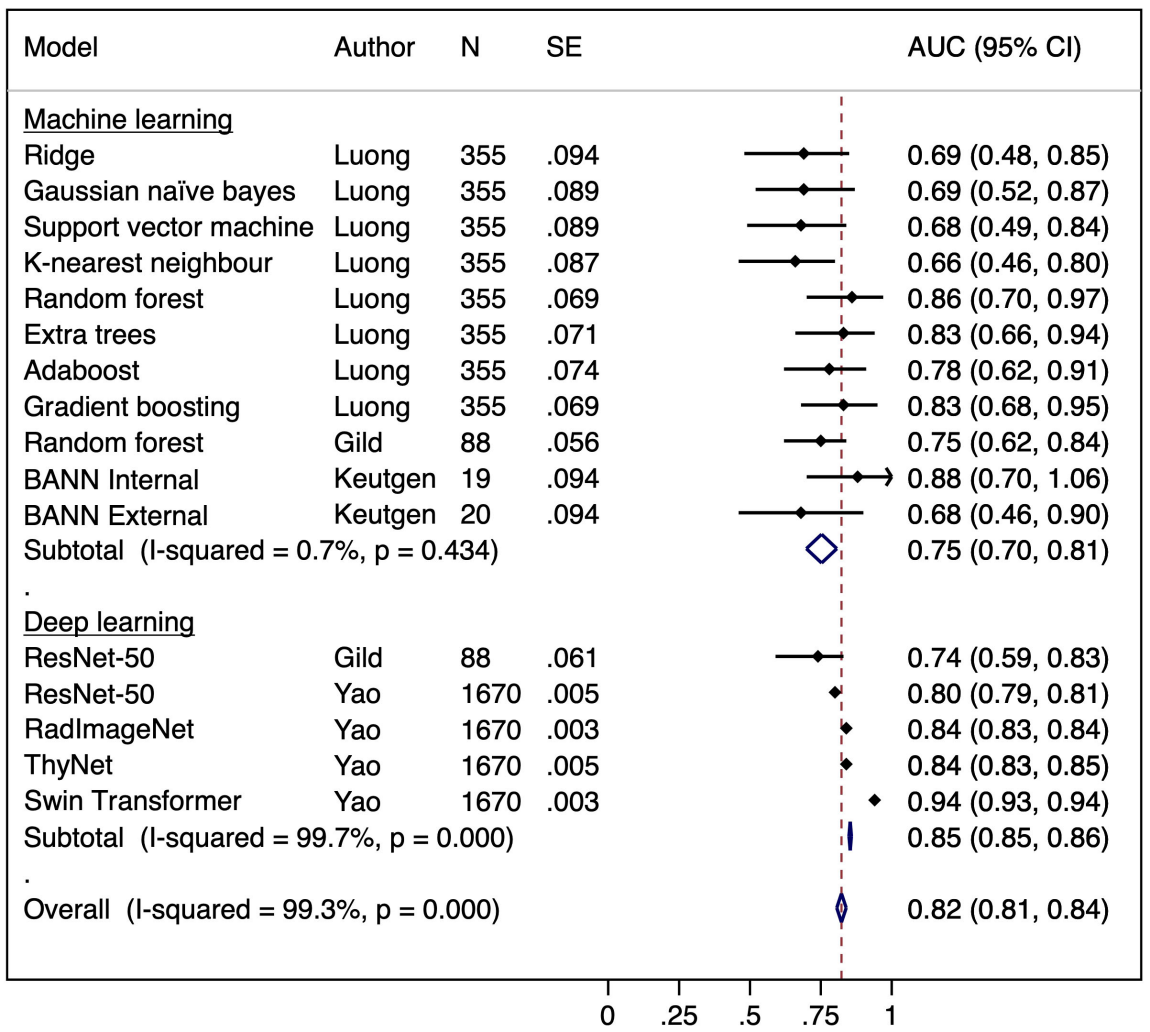
Forest plot of a random effects meta-analysis of area under the curve (AUC) for the observed AI models predicting malignancy in indeterminate cytology thyroid nodules. BANN, Bayesian artificial neural network.

**Figure 3:**
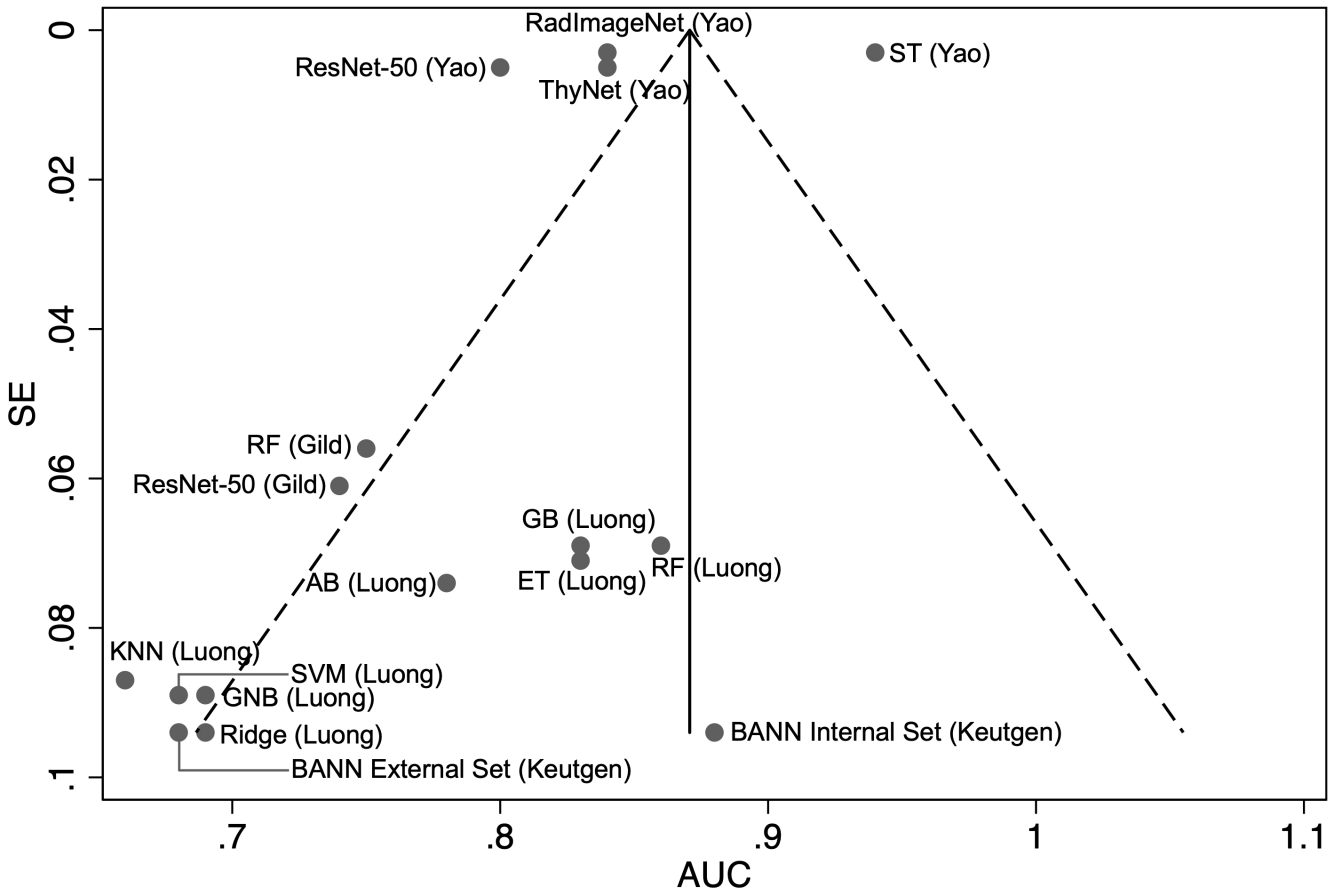
Funnel plot with pseudo 95% confidence limits of the area under the curve (AUC) versus the standard error (SE) for the each of the included studies (*N* = 16). RF, random forest; BANN, Bayesian artificial neural network; KNN, K-Nearest Neighbour; GNB, Gaussian Naïve Bayes; SVM, support vector machine; ET, Extra Trees; AB, AdaBoost; GB, gradient boosting.

Our pooled meta-analysis incorporated 16 AUC results derived from 15 distinct models across three studies. The AUC values from the two models presented by Gild et al. (34), the four models from Yao et al. (36), along with the eight models developed by Luong et al. (35), as well as the results of the model by Keutgen et al. (42), tested on two distinct cohorts, were combined for analysis (**Figure 2**). The model by Saini et al. (38) was excluded due to an AUC of 1.0 indicating perfect separation in a limited cohort (N = 11), which raises concerns regarding a meaningful estimate of real-world model discrimination. The pooled analysis of AUC across studies yielded a combined estimate of 0.82 (95% CI: 0.81–0.84) indicating moderate to good classification performance across ML and DL models (**Figure 2**). However, there is considerable heterogeneity among the studies, as indicated by a calculated I² value of 99.3%. The funnel plot was asymmetrical (**Figure 3**).

#### 3.7.3 Heterogeneity analysis

To further investigate the sources of heterogeneity, a subgroup analysis was conducted to compare the performance of ML and DL models. This revealed minimal heterogeneity in ML models (I² = 0.7%, pooled AUC = 0.75, 95% CI: 0.70–0.81), whereas DL models exhibited substantial heterogeneity (I² = 99.7%, pooled AUC = 0.85, 95% CI: 0.85–0.86) suggesting inconsistent performance. (**Figure 2**) Egger’s test was significant (p = 0.0006) indicating potential publication bias or systematic differences in study characteristics amongst DL models. Meta-regression found no significant association between SE and AUC (p = 0.568), suggesting that heterogeneity is not explained by study precision alone and may instead be influenced by differences in DL model architectures, dataset composition, or validation methodologies (33, 35).

### 3.8 Risk of bias assessment with the PROBAST

Overall, the PROBAST assessment revealed a “low risk of bias” for the studies by Keutgen et al. (42) and Yao et al. (36), while the other studies were judged to have an overall “high risk of bias” in one or more domains (**Table 2**). All studies exhibited an overall “low concern” for applicability to the review question. **Supplementary Material 3** provides a narrative risk of bias analysis for each study.

**Table 2:** Tabular presentation of PROBAST results.

Study	Risk of bias				Applicability			Overall	
	Participants	Predictors	Outcome	Analysis	Participants	Predictors	Outcome	Risk of bias	Applicability
Gild 2021(34)	−	+	−	−	+	+	+	−	+
Swan 2022(39)	+	−	+	+	+	+	+	−	+
Keutgen 2022(42)	+	+	+	+	+	+	+	+	+
Luong 2021(35)	+	+	+	−	+	+	+	−	+
Yao 2023(36)	+	+	+	+	+	+	+	+	+
Chen 2022(37)	−	−	+	−	+	+	+	−	+
Saini 2022(38)	−	−	−	−	+	+	?	−	+

PROBAST, Prediction model Risk Of Bias Assessment Tool; ROB, risk of bias.

+ indicates low ROB/low concern regarding applicability, − indicates high ROB/high concern regarding applicability, and? indicates unclear ROB/unclear concern regarding applicability.

## 4 Discussion

### 4.1 Main findings

The efficacy of AI tools for the pre-operative diagnosis of ITNs without the use of GSC was assessed by seven studies, five of which were radiologically driven, one cytology based, and one of which utilised NLP on unstructured EMR data. The 16 AI models suitable for meta-analysis had varying performances and accuracies, with a pooled AUC of 0.82. All included studies demonstrate the potential of AI to be of clinical value; however, there are limitations and substantial capacity for further development.

The externally validated AIBx model achieved an accuracy of 51% for all included nodules and 53% for ITNs, which restricts external institution clinical implementation currently based on performance alone (39). Similarly, the model developed by Keutgen et al. (42) demonstrated an internal validation AUC of 0.88 indicative of strong predictive performance within its own institution. However, this markedly decreased to 0.68 upon external validation suggesting a potential issue with overfitting. Overfitting occurs when a model performs exceptionally well on the training data but fails to generalise effectively to new, unseen datasets. This phenomenon can lead to inflated performance metrics during initial assessments, which may not reflect the model’s true applicability in clinical settings. Unlike studies that exhibit strong internal performance but degrade significantly upon external validation, Yao et al. (36) employed multicentre data within a 10-fold cross validation framework, rather than testing on an independent external dataset. As such, the consistently high AUCs reported in their study (AUC range: 0.80–0.94) may reflect the advantages of training and validation strategies rather than true external generalisability. While the study did compare model performance across independent test sets within their multicentre dataset, which provides some assessment of generalisability across institutions, this validation, however, was not structured to specifically evaluate performance in distinguishing benign from malignant cases across an entirely unseen cohort. These findings underscore the importance of considering dataset handling and model evaluation design when interpreting validation results.

A similar methodology was employed by Gild et al. (34), who also applied 10-fold cross validation but on a significantly smaller, single-centre dataset (N = 88). Their ResNet-50 model achieved an AUC of 0.740 (95% CI: 0.590–0.830) notably lower than the AUC of 0.803 (95% CI: 0.794–0.812) reported by Yao et al. (36) for the same architecture. This discrepancy likely reflects differences in dataset size and diversity, as the multicentre cohort provided greater heterogeneity and a larger sample for training. The greater performance variability in the results of Gild et al. suggests that their model was more susceptible to overfitting due to the limited dataset size. Yao et al. (36) also retrained ThyNet on their dataset achieving a higher AUC of 0.840 (95% CI: 0.834–0.846). In contrast, Gild et al. (34) tested it directly on their dataset, where it yielded an overall accuracy of 0.64. The lower performance of ThyNet in this setting suggests that, despite being specifically trained for thyroid imaging on 18,049 images, it struggled to generalise effectively when applied to an unseen dataset without adaptation.

It is notable that the Swin Transformer, despite being originally trained on a general purpose ImageNet-1000 (46) dataset, outperformed the institutionally fine-tuned ThyNet. This may be attributed to the Swin Transformer’s shifted window attention mechanism, which enhances the model’s ability to process medical images more effectively (47). The observed performance advantage aligns with our heterogeneity analysis, which suggests that variations in model architectures contribute significantly to differences in model performance. The lack of a significant association between SE and AUC in the meta-regression further reinforces this notion indicating that small-study effects do not fully explain the observed heterogeneity. Instead, systematic differences in DL architectures and methodological choices emerge as primary contributors. These findings emphasise the need for standardised evaluation frameworks and rigorous validation practices in AI research to enhance reproducibility.

The NLP-driven models produced a mean accuracy of 70% and mean AUC of 0.754 across all classifiers (35). Notably, the study indicated that echogenicity and calcification were of low feature importance in predicting malignancy, a finding that contradicts the established TIRADS criteria. This discrepancy may be attributable to the high rate of missingness associated with these two variables, thereby limiting the generalisability of the results. Data imputation techniques were utilised to populate the missing values of these categorical variables. However, echogenicity and calcification had a missing rate of 99% and 88%, respectively, and in such high proportions of incomplete data, estimates are likely to be biased.

Both Saini et al. (38) and Chen et al. (37) rely on manually assessed imaging or cytological parameters making their models prone to inter-reader variability and limiting reproducibility. The ANN is trained on semi-quantitative cytological features, which depend on subjective grading by independent observers, introducing variability in how key predictors are assessed (38). Chen et al. (37) similarly employs an SVM model trained on USG features manually evaluated by radiologists. The retrospective reassessment of included images, all obtained using high-frequency linear transducers, potentially leads to inconsistencies in real-world applications. Since neither study incorporates automated feature extraction, their performance may vary across institutions and readers with different expertise levels. Without independent validation, the generalisability of these models remains uncertain. Future DL approaches that extract imaging features directly from raw data could enhance clinical applicability by reducing dependence on subjective interpretation.

### 4.2 Related works

Swan et al. (39) externally validated AIBx, which was developed in 2019 at Mercy Hospital, USA, utilising USG images of thyroid nodules obtained from patients who underwent biopsy or thyroid surgery between February 2012 and February 2017. Only nodules with a definitive diagnosis of benign or malignant were included in AIBx’s construct. A total of 482 nodules fulfilled the inclusion criteria, with all available images used to create their image similarity AI model. The architecture comprised of a 34-layer CNN known as ResNet-34. The CNN generated image embeddings, which are *N*-dimensional vectors representing unique images. These embeddings were used to find similar images from a database using a nearest neighbour algorithm. The output includes *N* number of nearest neighbours along with their corresponding labels of benign vs. malignant (48, 49). The model was internally validated using 103 thyroid nodules that underwent biopsy or surgery from March 2017 to July 2018. Accuracy, sensitivity, specificity, PPV and NPV of the model were 81.5%, 87.8%, 78.5%, 65.9%, and 93.2%, respectively (40). Compared to USG thyroid cancer risk stratification systems, AIBx exhibited comparable performance suggesting that from an institutional perspective, the model has the potential to avoid unnecessary FNAC (5, 6).

Similar to AIBx, ThyNet was designed as a strategy to help radiologists avoid unnecessary FNAC. Its structure is an integrated network of ResNet (50), ResNext (51), and DenseNet (50), which, when evaluated individually on internal validation sets, achieved AUCs of 0.9376, 0.9348, and 0.9401, respectively, in classifying nodules into benign or malignant. After model ensemble, the AUC achieved was 0.9504, which outperformed any one individual model. In a simulated scenario, a radiologist assisted by ThyNet strategy was reported to decrease the number of FNAC from 61.9% to 35.2%, and the missed malignancy rate decreased from 18.9% to 17.0%. In the real-world clinical setting test of ThyNet, the AUC of a thyroid nodule diagnosis, where radiologists reviewed static images only, was 0.823 (95% CI 0.812–0.835); the AUC of a diagnosis where radiologists reviewed both videos and images improved to 0.862 (0.851–0.872; p < 0.0001); and finally, when radiologists were assisted by ThyNet, the AUC improved to 0.873 (0.863–0.883; p < 0.0001) (41). These findings suggest that the ThyNet system could potentially be used to complement the decision-making process of FNAC alongside radiologists; however, as a stand-alone diagnostic system for Bethesda III nodules, it has restricted applicability (34).

The clinical narrative has unique characteristics different from other forms of literature and text. NLP within healthcare leverages this distinctive lexicon, and these models are trained to extract precise information from large amounts of unstructured clinical text while considering contextual factors. This form of language-based AI has been explored in aiding the interpretation of thyroid USG reports as these can be rather challenging due to the lack of standardised synoptic reporting despite the TIRADS score. In two studies by the same group, USG reports were interpretated by clinicians as a gold standard and compared with NLP data extraction using cTAKES. Results suggest the need for improved synoptic reporting of thyroid USG, as NLP was effective in automated extraction of data from USG reports; however, the lack of standardised synoptic reporting caused a significant difference between gold standard and NLP performance (43, 52, 53).

### 4.3 Limitations

A meta-analysis for the performance AI models in healthcare presents inherent challenges. Traditional meta-analysis requires studies to have similar interventions; however, at present, AI research attempts to investigate intrinsically different model architectures and their optimal applicability tested on identical patient cohorts. These difficulties are a result of data scarcity, early-stage research, and a presently evolving landscape. However, to provide a synthesised inference of the current available evidence, we did conduct a meta-analysis. Additionally, there are limitations to AI tools in healthcare apart from a model’s performance compared with a human expert. The ability for widespread application and adaptability is a major drawback. This challenge arises from a dataset bias, as most AI models are trained on a single institution or hubs’ data. Once trained on a particular demographic, AI models tend to lose their diversity in transferability and are unable to perform as well in an external setting similar to the findings from the external validation of AIBx and ThyNet (34, 39).

Informed consent is significant in decision-making tools. AI as a “black box,” however, presents a narrative challenge. It is a difficult and time-consuming effort to explicate a process that lacks a state of explainability. Radiologically driven models in this review generally lacked meaningful decision-making interpretability or model uncertainty assessments. Heat maps were explored alongside image classification algorithms but were often unhelpful for clinical decision support. Alternative approaches, such as image similarity algorithms, have provided more intuitive interfaces for clinicians allowing them to review matched USG images. Yet, the underlying indexing mechanisms remain opaque (40). Feature energy mapping has also been investigated as a way to visualise model attention, but without clear correlation to established radiological markers, its clinical relevance remains uncertain (36). A potential solution to these challenges is the integration of interactive interpretability frameworks, such as those used in ThyGPT, which allows clinicians to query AI-generated heat maps, adjust inputs, and observe changes in diagnostic predictions (54). It additionally incorporates language models that generate structured explanations based on clinical guidelines. While this does not fully resolve model opacity, it improves clinician oversight and aligns AI interpretations more closely with expert reasoning.

## 5 Conclusion

This review highlights the current lack of clinically applicable evidence to support the reliable pre-operative diagnosis of ITNs using AI. These tools have a potential role in the risk stratification of thyroid nodules and are in their early stages of establishment. There is a need to investigate the generalisability of models created, as the majority are developed and tested within an institutional setting. Consideration must also be given to ethical issues and trust surrounding the use of AI in healthcare.

## Data Availability

The original contributions presented in the study are included in the article/**Supplementary Material**. Further inquiries can be directed to the corresponding author.
