# Supplementary material for: Beyond genomics: artificial intelligence-powered diagnostics for indeterminate thyroid nodules—a systematic review and meta-analysis
Source: Front Endocrinol (Lausanne). 2025 May 5;16:1506729. doi: 10.3389/fendo.2025.1506729 (PMC12086071; doi:10.3389/fendo.2025.1506729)
Supplement: Supplementary file 1 [file DataSheet1.docx]

**Supplemental Table 1**. List of studies screened

| **Author (Year)** | **Title** | **Eligibility** |
| --- | --- | --- |
| Al Hakim (2021) | Artificial Intelligence for Thyroid Disorders: A Systematic Review | EXCLUDE on evidence |
| Alabrak (2023) | Artificial intelligence role in subclassifying cytology of thyroid follicular neoplasm | EXCLUDE on target group |
| Assaad (2023) | Thyroid cytopathology cancer diagnosis from smartphone images using machine learning | EXCLUDE on target group |
| Boers (2023) | Ultrasound imaging in thyroid nodule diagnosis, therapy, and follow‐up: Current status and future trends | EXCLUDE on evidence |
| Boruah (2024) | Modern Thyroid Cancer Diagnosis: A Review of AI-Powered Algorithms for Detection and Classification. | EXCLUDE on evidence |
| Cantisani (2020) | Artificial intelligence: what is it and how can it expand the ultrasound potential in the future? | EXCLUDE on evidence |
| Cao (2023) | Artificial intelligence in thyroid ultrasound | EXCLUDE on target group |
| Chai (2020) | Artificial intelligence for thyroid nodule ultrasound image analysis | EXCLUDE on evidence |
| Chen (2022) | Machine Learning–Assisted Diagnostic System for Indeterminate Thyroid Nodules | INCLUDE |
| Chen (2023) | Diagnostic value of American College of Radiology Thyroid Imaging Reporting and Data System combined with elastography in differentiating clinically atypical subacute thyroiditis from papillary thyroid carcinoma: a single retrospective research | EXCLUDE on intervention |
| Chen (2024) | Assessing the feasibility of ChatGPT-4o and Claude 3-Opus in thyroid nodule classification based on ultrasound images | EXCLUDE on target group |
| Chen (2025) | A multicenter diagnostic study of thyroid nodule with Hashimoto’s thyroiditis enabled by Hashimoto’s thyroiditis nodule-artificial intelligence model | EXCLUDE on target group |
| Choi (2010) | Interobserver and intraobserver variations in ultrasound assessment of thyroid nodules | EXCLUDE on intervention |
| Choi (2017) | A computer-aided diagnosis system using artificial intelligence for the diagnosis and characterization of thyroid nodules on ultrasound: initial clinical assessment | EXCLUDE on target group |
| Cibas (2009) | The Bethesda system for reporting thyroid cytopathology | EXCLUDE on evidence |
| Cordes (2023) | Ultrasound characteristics of follicular and parafollicular thyroid neoplasms: diagnostic performance of artificial neural network | EXCLUDE on target group |
| D’Andréa (2023) | Application of machine learning methods to guide patient management by predicting the risk of malignancy of Bethesda III-V thyroid nodules | EXCLUDE on evidence |
| Daniels (2020) | Machine learning by ultrasonography for genetic risk stratification of thyroid nodules | EXCLUDE on target group |
| David (2024) | Thyroid nodule characterization: Overview and state of the art of diagnosis with recent developments, from imaging to molecular diagnosis and artificial intelligence | EXCLUDE on evidence |
| Diuldin (2024) | Using Deep Learning to Generate and Classify Thyroid Cytopathology Reports According to The Bethesda System. | EXCLUDE on target group |
| Dong (2022) | Establishment and validation of a radiological-radiomics model for predicting high-grade patterns of lung adenocarcinoma less than or equal to 3 cm | EXCLUDE on target group |
| Dov (2022) | Use of machine learning–based software for the screening of thyroid cytopathology whole slide images | EXCLUDE on target group |
| Dov (2023) | Deep-Learning–Based Screening and Ancillary Testing for Thyroid Cytopathology | EXCLUDE on target group |
| Duc (2022) | An ensemble deep learning for automatic prediction of papillary thyroid carcinoma using fine needle aspiration cytology | EXCLUDE on target group |
| Duinkerken (2021) | Jefferson Digital Commons quarterly report: January-March 2021 | EXCLUDE on evidence |
| Elliott Range (2020) | Application of a machine learning algorithm to predict malignancy in thyroid cytopathology | EXCLUDE on target group |
| Farrag (2006) | Importance of routine evaluation of the thyroid gland prior to open partial laryngectomy | EXCLUDE on target group |
| Fiorentino (2023) | The minefield of indeterminate thyroid nodules: could artificial intelligence be a suitable diagnostic tool? | EXCLUDE on evidence |
| Fragopoulos (2020) | Radial basis function artificial neural network for the investigation of thyroid cytological lesions | EXCLUDE on evidence |
| Gao (2018) | Computer‐aided system for diagnosing thyroid nodules on ultrasound: A comparison with radiologist‐based clinical assessments | EXCLUDE on target group |
| Gibelin (2005) | Increased calcitonin level in thyroid nodules without medullary carcinoma | EXCLUDE on target group |
| Gild (2022) | Risk stratification of indeterminate thyroid nodules using ultrasound and machine learning algorithms. | INCLUDE |
| Girolami (2020) | Impact of image analysis and artificial intelligence in thyroid pathology, with particular reference to cytological aspects | EXCLUDE on evidence |
| Guerrisi (2024) | A Machine Learning Model Based on Thyroid US Radiomics to Discriminate Between Benign and Malignant Nodules | EXCLUDE on target group |
| Gulcelik (2008) | Risk of malignancy in patients with follicular neoplasm: predictive value of clinical and ultrasonographic features | EXCLUDE on intervention |
| Guo (2023) | Assessment of the Statistical Optimization Strategies and Clinical Evaluation of an Artificial Intelligence–Based Automated Diagnostic System for Thyroid Nodule Screening | EXCLUDE on target group |
| Guo (2023) | Assessment of the statistical optimization strategies and clinical evaluation of an artificial intelligence-based automated diagnostic system for thyroid nodule screening | EXCLUDE on target group |
| Ha (2024) | Artificial intelligence model assisting thyroid nodule diagnosis and management: a multicenter diagnostic study | EXCLUDE on target group |
| Hales (2006) | S071 Is There a Role for FDG-PET/CT in Cytologically Indeterminate Thyroid Nodules? | EXCLUDE on intervention |
| Hong (2019) | Diagnostic performance of ultrasound patterns by K-TIRADS and 2015 ATA guidelines in risk stratification of thyroid nodules and follicular lesions of undetermined significance | EXCLUDE on intervention |
| Huang (2023) | Concordance of the ACR TI-RADS classification with bethesda scoring and histopathology risk stratification of thyroid nodules | EXCLUDE on evidence |
| Idarraga (2021) | False negative rates in benign thyroid nodule diagnosis: machine learning for detecting malignancy | EXCLUDE on target group |
| Jassal (2023) | Artificial Intelligence for Pre-operative Diagnosis of Malignant Thyroid Nodules Based on Sonographic Features and Cytology Category | EXCLUDE on target group |
| Jassal (2024) | Attention‐based image segmentation and classification model for the preoperative risk stratification of thyroid nodules | EXCLUDE on target group |
| Jin (2024) | Cytologic assessment of thyroid nodules–Updates in 2023 Bethesda reporting system, diagnostic challenges and pitfalls | EXCLUDE on evidence |
| Keleşoğlu (2024) | Investigating the Applicability of the McGill Thyroid Nodule Score (MTNS) in Patients Undergoing Surgery for Thyroid Nodules: A Comparison Between Patients with and Without Papillary Thyroid Cancer. | EXCLUDE on evidence |
| Keutgen (2022) | A machine-learning algorithm for distinguishing malignant from benign indeterminate thyroid nodulesusing ultrasound radiomic features | INCLUDE |
| Kezlarian (2021) | Artificial intelligence in thyroid fine needle aspiration biopsies | EXCLUDE on target group |
| Kim (2013) | Color Doppler features of solid, round, isoechoic thyroid nodules without malignant sonographic features: a prospective cytopathological study | EXCLUDE on intervention |
| Komatsu (2021) | Towards clinical application of artificial intelligence in ultrasound imaging | EXCLUDE on target group |
| Lassau (2019) | Five simultaneous artificial intelligence data challenges on ultrasound, CT, and MRI | EXCLUDE on target group |
| Lee (2024) | Improved diagnostic accuracy of thyroid fine-needle aspiration cytology with artificial intelligence technology | EXCLUDE on evidence |
| Lee (2025) | A Machine Learning-Based Radiomics Model for the Differential Diagnosis of Benign and Malignant Thyroid Nodules in F-18 FDG PET/CT: External Validation in the Different Scanner | EXCLUDE on target group |
| Liang (2019) | Update on thyroid ultrasound: a narrative review from diagnostic criteria to artificial intelligence techniques | EXCLUDE on evidence |
| Liang (2020) | Convolutional neural network for breast and thyroid nodules diagnosis in ultrasound imaging | EXCLUDE on target group |
| Lin (2019) | Computer-aided diagnostic technique in 2-deoxy-2-[18F] fluoro-d-glucose-positive thyroid nodule: clinical experience of 74 non-thyroid cancer patients | EXCLUDE on intervention |
| Lin (2021) | Deep learning fast screening approach on cytological whole slides for thyroid cancer diagnosis | EXCLUDE on target group |
| LIN (2023) | Research advances in artificial intelligence in cytopathologic diagnosis of thyroid | EXCLUDE on evidence |
| Liu (2023) | The auxiliary diagnosis of thyroid echogenic foci based on a deep learning segmentation model: A two-center study | EXCLUDE on target group |
| Loss (2019) | JAMA Otolaryngology–Head & Neck Surgery | EXCLUDE on target group |
| Lozhkin (2024) | Features of Intelligent Processing of Cytological Whole Slide Images | EXCLUDE on target group |
| Lu (2023) | Three-dimensional ultrasound-based radiomics nomogram for the prediction of extrathyroidal extension features in papillary thyroid cancer | EXCLUDE on target group |
| Ludwig (2023) | The use of artificial intelligence in the diagnosis and classification of thyroid nodules: an update | EXCLUDE on evidence |
| Luong (2022) | Risk Stratifying Indeterminate Thyroid Nodules With Machine Learning. | INCLUDE |
| Ma (2020) | Efficient deep learning architecture for detection and recognition of thyroid nodules | EXCLUDE on target group |
| Ma (2024) | A study of machine learning models for rapid intraoperative diagnosis of thyroid nodules for clinical practice in China | EXCLUDE on target group |
| Manelli (2022) | Deep Learning-Based Artificial Intelligence to Predict Cancer in Cytologically Indeterminate Thyroid Nodules: A multi-centre European study | EXCLUDE on evidence |
| Maweni (2022) | Malignancy rates and initial management of Thy3 thyroid nodules in a district general hospital: The ‘Reading’experience | EXCLUDE on intervention |
| McQueen (2018) | Head and neck ultrasound: technical advances, novel applications and the role of elastography | EXCLUDE on target group |
| Mitchell (2019) | Decision Making in Indeterminate Thyroid Nodules and the Role of Molecular Testing | EXCLUDE on intervention |
| Na (2016) | Thyroid imaging reporting and data system risk stratification of thyroid nodules: categorization based on solidity and echogenicity | EXCLUDE on target group |
| Nair (2024) | Combining Image Similarity and Predictive Artificial Intelligence Models to Decrease Subjectivity in Thyroid Nodule Diagnosis and Improve Malignancy Prediction | EXCLUDE on target group |
| Nguyen (2019) | Artificial intelligence-based thyroid nodule classification using information from spatial and frequency domains | EXCLUDE on target group |
| Nguyen (2020) | Ultrasound image-based diagnosis of malignant thyroid nodule using artificial intelligence | EXCLUDE on target group |
| Orloff (2020) | Thyroid ultrasound: Machine beats humans at detecting malignant nodules | EXCLUDE on target group |
| Ozturk (2023) | Differentiation of Benign and Malignant Thyroid Nodules with ANFIS by Using Genetic Algorithm and Proposing a Novel CAD-Based Risk Stratification System of Thyroid Nodules | EXCLUDE on intervention |
| Park (2021) | Artificial intelligence for ultrasonography: unique opportunities and challenges | EXCLUDE on evidence |
| Peng (2021) | Deep learning-based artificial intelligence model to assist thyroid nodule diagnosis and management: a multicentre diagnostic study | EXCLUDE on target group |
| Poursina (2025) | Artificial Intelligence and Whole Slide Imaging Assist in Thyroid Indeterminate Cytology: A Systematic Review | EXCLUDE on evidence |
| Rao (2024) | Assessment of the risk of malignancy in Bethesda III thyroid nodules: a comprehensive review | EXCLUDE on evidence |
| Ren (2024) | Dual-modal radiomics nomogram based on contrast-enhanced ultrasound to improve differential diagnostic accuracy and reduce unnecessary biopsy rate in ACR TI-RADS 4–5 thyroid nodules | EXCLUDE on target group |
| Rizzo (2024) | The application of artificial intelligence to thyroid nodule assessment | EXCLUDE on target group |
| Ryu (2024) | Differential diagnosis of thyroid nodules using heterogeneity quantification software on ultrasound images: correlation with the Bethesda system and surgical pathology | EXCLUDE on target group |
| Saini (2023) | An artificial neural network for the prediction of the risk of malignancy in category III Bethesda thyroid lesions | INCLUDE |
| Sant (2024) | From Bench-to-Bedside: How Artificial Intelligence is Changing Thyroid Nodule Diagnostics, a Systematic Review | EXCLUDE on evidence |
| Sanyal (2018) | Artificial intelligence in cytopathology: a neural network to identify papillary carcinoma on thyroid fine-needle aspiration cytology smears | EXCLUDE on target group |
| Saoud (2024) | The Bethesda System for Reporting Thyroid Cytopathology in the African American population: A tertiary centre experience | EXCLUDE on evidence |
| Sauter (2019) | Assessment of The Bethesda System for Reporting Thyroid Cytopathology: Surgical and long-term clinical follow-up of 2,893 thyroid fine-needle aspirations | EXCLUDE on evidence |
| Schumm (2023) | Prognostic value of preoperative molecular testing and implications for initial surgical management in thyroid nodules harboring suspected (Bethesda V) or known (Bethesda VI) papillary thyroid cancer | EXCLUDE on evidence |
| Shah (2010) | Decision making for the extent of thyroidectomy in the patient with atypical cytologic results | EXCLUDE on intervention |
| Shen (2021) | Artificial intelligence in ultrasound | EXCLUDE on target group |
| Slabaugh (2023) | Applications of machine and deep learning to thyroid cytology and histopathology: a review | EXCLUDE on evidence |
| Słowińska-Klencka (2021) | Validation of Four Thyroid Ultrasound Risk Stratification Systems in Patients with Hashimoto’s Thyroiditis; Impact of Changes in the Threshold for Nodule’s Shape Criterion | EXCLUDE on target group |
| Song (2019) | Ultrasound image analysis using deep learning algorithm for the diagnosis of thyroid nodules | EXCLUDE on target group |
| Stojadinovic (2009) | Development of a clinical decision model for thyroid nodules | EXCLUDE on target group |
| Swan (2022) | External validation of AIBx, an artificial intelligence model for risk stratification, in thyroid nodules | INCLUDE |
| Taccogna (2022) | An innovative synthetic support for immunocytochemical assessment of cytologically indeterminate (Bethesda III) thyroid nodules | EXCLUDE on evidence |
| Tahmasebi (2021) | Assessment of axillary lymph nodes for metastasis on ultrasound using artificial intelligence | EXCLUDE on target group |
| Takeyama (2009) | Sentinel node biopsy for follicular tumours of the thyroid gland | EXCLUDE on target group |
| Tama (2020) | Recent advances in the application of artificial intelligence in otorhinolaryngology-head and neck surgery | EXCLUDE on evidence |
| Thomas (2020) | AIBx, artificial intelligence model to risk stratify thyroid nodules | EXCLUDE on target group |
| Thomas (2020) | Use of artificial intelligence and machine learning for estimating malignancy risk of thyroid nodules | EXCLUDE on evidence |
| Underwood (2018) | Revisiting the 2015 American Thyroid Association Guidelines With Respect to Indeterminate Thyroid Nodules in the Era of Noninvasive Follicular Thyroid Neoplasm With Papillary-like Nuclear Features | EXCLUDE on evidence |
| Verburg (2019) | Sonographic diagnosis of thyroid cancer with support of AI | EXCLUDE on target group |
| Villanueva (unknown) | Machine Learning Classifier for Preoperative Diagnosis of Benign Thyroid Nodules | EXCLUDE on target group |
| Wamkpah (2021) | Patients’ views of shared decision-making and decisional conflict in otolaryngologic surgery during the COVID-19 pandemic | EXCLUDE on target group |
| Wang (2019) | Automatic thyroid nodule recognition and diagnosis in ultrasound imaging with the YOLOv2 neural network | EXCLUDE on target group |
| Wang (2019) | Artificial intelligence in ultrasound imaging: Current research and applications | EXCLUDE on evidence |
| Wang (2020) | Comparison study of radiomics and deep learning-based methods for thyroid nodules classification using ultrasound images | EXCLUDE on target group |
| Wang (2023) | Diagnostic value of a dynamic artificial intelligence ultrasonic intelligent auxiliary diagnosis system for benign and malignant thyroid nodules in patients with Hashimoto thyroiditis | EXCLUDE on target group |
| Wei (2020) | Visual interpretability in computer-assisted diagnosis of thyroid nodules using ultrasound images | EXCLUDE on target group |
| Wei (2021) | Radiomics based on multiparametric MRI for extrathyroidal extension feature prediction in papillary thyroid cancer | EXCLUDE on target group |
| Wildman-Tobriner (2019) | Using artificial intelligence to revise ACR TI-RADS risk stratification of thyroid nodules: diagnostic accuracy and utility | EXCLUDE on target group |
| Wildman-Tobriner (2024) | Simplifying risk stratification for thyroid nodules on ultrasound: validation and performance of an artificial intelligence thyroid imaging reporting and data system | EXCLUDE on target group |
| Wong (2023) | Current status of machine learning in thyroid cytopathology | EXCLUDE on evidence |
| Wu (2014) | Mobile wound assessment using novel computer vision methods | EXCLUDE on target group |
| Wu (2022) | Radiomics Analysis of Computed Tomography for Prediction of Thyroid Capsule Invasion in Papillary Thyroid Carcinoma: A Multi-Classifier and Two-Center Study | EXCLUDE on target group |
| Wu (2023) | Digital Health for Patients Undergoing Cardiac Surgery: A Systematic Review. Healthcare 2023, 11, 2411 | EXCLUDE on target group |
| Wylie (2016) | Molecular classification of thyroid lesions by combined testing for miRNA gene expression and somatic gene alterations. | EXCLUDE on target group |
| Xia (2019) | A computer-aided diagnosing system in the evaluation of thyroid nodules—experience in a specialized thyroid center | EXCLUDE on target group |
| Xu (2022) | Value of Whole-Thyroid CT-Based Radiomics in Predicting Benign and Malignant Thyroid Nodules | EXCLUDE on target group |
| Xu (2023) | The Performance of Deep Learning on Thyroid Nodule Imaging Predicts Thyroid Cancer: A Systematic Review and Meta-Analysis of Epidemiological Studies with Independent External Test Sets | EXCLUDE on evidence |
| Xu (2024) | Automatic detection of thyroid nodules with a real-time artificial intelligence system in a real clinical scenario and the associated influencing factors | EXCLUDE on target group |
| Yaghoubi (2023) | Investigation of Clinical Features and Surgical Results in Patients with Thyroid Nodules Referred to the Endocrinology Clinic in Hamadan City | EXCLUDE on target group |
| Yang (2024) | A narrative review of deep learning in thyroid imaging: current progress and future prospects | EXCLUDE on evidence |
| Yao (2022) | A study of thyroid fine needle aspiration of follicular adenoma in the “atypia of undetermined significance” Bethesda category using digital image analysis | EXCLUDE on target group |
| Yao (2023) | AI diagnosis of Bethesda category IV thyroid nodules | INCLUDE |
| Yoon (2020) | Artificial intelligence to predict the BRAFV600E mutation in patients with thyroid cancer | EXCLUDE on target group |
| Zhong (2024) | Combined model integrating clinical, radiomics, BRAFV600E and ultrasound for differentiating between benign and malignant indeterminate cytology (Bethesda III) thyroid nodules: a bi-center retrospective study | EXCLUDE on evidence |
| Zhou (2020) | 2020 Chinese guidelines for ultrasound malignancy risk stratification of thyroid nodules: the C-TIRADS | EXCLUDE on evidence |
| Zhu (2019) | Deep neural networks could differentiate Bethesda class III versus class IV/V/VI | EXCLUDE on target group |
| یعقوبی (2023) | بررسی ویژگی‌های بالینی و نتایج جراحی در بیماران مبتلا به ندول‌های تیروئیدی مراجعه‌کننده به کلینیک غدد شهر همدان | EXCLUDE on language |
| 김극배 (1991) | 갈색세포종 | EXCLUDE on language |
| 林宇 (2023) | 人工智能在甲状腺细胞病理学诊断中的研究进展 | EXCLUDE on language |
