# Supplementary material for: Beyond genomics: artificial intelligence-powered diagnostics for indeterminate thyroid nodules—a systematic review and meta-analysis
Source: Front Endocrinol (Lausanne). 2025 May 5;16:1506729. doi: 10.3389/fendo.2025.1506729 (PMC12086071; doi:10.3389/fendo.2025.1506729)
Supplement: Supplementary file 2 [file DataSheet2.docx]

**Supplemental Material 2.** Database search strategy

| **#** | **Searches** |
| --- | --- |
| 1 | Preoperative/ or Preoperative Diagnosis |
| 2 | Evaluation/ or Assessment/ or Diagnosis |
| 3 | Artificial Intelligence/ or Machine Learning |
| 4 | Indeterminate thyroid nodule/ or Indeterminate cytology |
| 5 | -molecular |
| 6 | Thyroid Nodule/pathology or Biopsy, Fine-Needle/methods |
| 7 | (1 AND 2) and (3) and (4) and (5) |
| 8 | (6) and (3) |
| 9 | exp animals/ not humans.sh. |
| 10 | 7 and 8 not 7 |
| 11 | Limit 10 to English language |

- Search performed by Dr Karishma Jassal
- Search performed from inception until February 18^th^ 2025
- Reference list of reviews and relevant papers hand searched for relevant studies
- Search results uploaded into ‘Eppi Reviewer 4’ and screened
- Same search strategy with minor search system alterations used for PUBMED- EMBASE + MEDLINE, GOOGLE SCHOLAR and SCOPUS
- Boolean string: preop* AND (diagno* OR evaluat*) AND ("artificial intelligence" OR "machine learning") AND "indeterminate thyroid nodules" -molecular
- Boolean string: ("thyroid nodule/pathology" OR "biopsy, fine-needle/methods") AND ("artificial Intelligence" OR "machine learning")
