# Supplementary material for: Beyond genomics: artificial intelligence-powered diagnostics for indeterminate thyroid nodules—a systematic review and meta-analysis
Source: Front Endocrinol (Lausanne). 2025 May 5;16:1506729. doi: 10.3389/fendo.2025.1506729 (PMC12086071; doi:10.3389/fendo.2025.1506729)
Supplement: Supplementary file 3 [file DataSheet3.docx]

**Supplemental material 3.** PROBAST Risk of Bias detailed explanation

Gild et al (Development, Validation study): The PROBAST risk of bias assessment revealed an overall ‘high risk of bias’. This arose from domain 1 and 3 (participants and outcomes) as “other” cancer types were included in the study and domain 4 (analysis) image pre-processing steps was not reported for the developed model and a low number of training images. There was also a lack of clarity regarding the training versus testing cohort in their reporting of Random Forest classifier results.

Swan et al (Validation study): Risk of bias assessment in this study was judged as an overall ‘high risk of bias’. Domain 2 (predictors) presents a risk of bias from concerns related to the first AIBx image displayed taken as the predictive diagnosis.

Keutgen et al (Development, Validation study): The study demonstrated a ‘low risk of bias’ across all PROBAST domains, with well-defined participants, predictors, and outcomes, and appropriate statistical analysis.

Luong et al (Development study): Risk of bias assessment for this study revealed an overall ‘high risk of bias’. This arose from domain 4 relating to high rates of missing data for certain features despite the use of imputation techniques, introducing potential selection bias.

Yao et al (Development, Validation study): The study demonstrated a ‘low risk of bias’ across all PROBAST domains, with well-defined participants, predictors, and outcomes, and appropriate statistical analysis.

Saini et al (Development, Validation study): The PROBAST risk of bias assessment revealed an overall high risk of bias. Domain 1 was at high risk due to the small sample size, which limits generalisability. Domain 3 had concerns as some benign cases were determined based on follow-up rather than definitive histopathology. Domain 4 was problematic because there was no independent external validation of the artificial neural network, increasing the risk of overfitting. Additionally, within domain 2 there are concerns regarding the reproducibility of the subjective cytological assessment used to develop the ANN model, as features were graded semi-quantitatively by two independent observers.

Chen et al (Development, Validation study): The PROBAST risk of bias assessment revealed an overall high risk of bias. Domain 1 was at high risk as the study was conducted at a single institution, limiting generalisability. Domain 2 was at high risk due to the subjective nature of radiologists retrospectively reassessing ultrasound images and reporting parameters, which may affect reproducibility. Domain 4 was also at high risk as there was no external validation, increasing the potential for overfitting and limiting applicability to broader clinical settings.
